# Assessing the suitability of a one‐time sampling event for close‐kin mark‐recapture: A caribou case study

**DOI:** 10.1002/ece3.70230

**Published:** 2024-09-03

**Authors:** Brandon D. Merriell, Micheline Manseau, Paul J. Wilson

**Affiliations:** ^1^ Environmental and Life Sciences Department Trent University Peterborough Ontario Canada; ^2^ Landscape Science and Technology Division, Environment and Climate Change Canada Ottawa Ontario Canada

**Keywords:** abundance estimation, caribou, close‐kin mark–recapture, non‐invasive sampling, *Rangifer tarandus*, simulation study, small populations, terrestrial systems

## Abstract

Abundance estimation is frequently an objective of conservation and monitoring initiatives for threatened and other managed populations. While abundance estimation via capture–mark–recapture or spatially explicit capture–recapture is now common, such approaches are logistically challenging and expensive for species such as boreal caribou (*Rangifer tarandus*), which inhabit remote regions, are widely dispersed, and exist at low densities. Fortunately, the recently developed ‘close‐kin mark–recapture’ (CKMR) framework, which uses the number of kin pairs obtained within a sample to generate an abundance estimate, eliminates the need for multiple sampling events. As a result, some caribou managers are interested in using this method to generate an abundance estimate from a single, non‐invasive sampling event for caribou populations. We conducted a simulation study using realistic boreal caribou demographic rates and population sizes to assess how population size and the proportion of the population surveyed impact the accuracy and precision of single‐survey CKMR‐based abundance estimates. Our results indicated that abundance estimates were biased and highly imprecise when very small proportions of the population were sampled, regardless of the population size. However, the larger the population size, the smaller the required proportion of the population surveyed to generate both accurate and reasonably precise estimates. Additionally, we also present a case study in which we used the CKMR framework to generate annual female abundance estimates for a small caribou population in Jasper National Park, Alberta, Canada, from 2006 to 2015 and compared them to existing published capture–mark–recapture‐based estimates. Both the accuracy and precision of the annual CKMR‐based abundance estimates varied across years and were sensitive to the proportion of pairwise kinship comparisons which yielded a mother–offspring pair. Taken together, our study demonstrates that it is possible to generate CKMR‐based abundance estimates from a single sampling event for small caribou populations, so long as a sufficient sampling intensity can be achieved.

## Graphical abstract

We conducted a simulation study using realistic boreal caribou demographic rates and population sizes to assess how population size and the proportion of the population surveyed impact the accuracy and precision of single‐survey CKMR‐based abundance estimates. Our results indicated that abundance estimates were biased and highly imprecise when very small proportions of the population were sampled, regardless of the population size; however, the larger the population size, the smaller the required proportion of the population surveyed to generate both accurate and reasonably precise estimates. We also present a case study in which we used the CKMR framework to generate annual female abundance estimates for a small caribou population in Jasper National Park, Alberta, Canada, from 2006 to 2015 and compared them to existing published capture–mark–recapture‐based estimates.

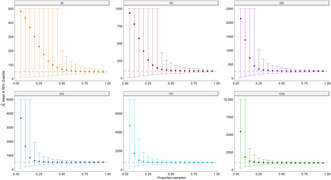

## 1 INTRODUCTION

Sound wildlife management and conservation rest on having reliable estimates of demographic parameters, including abundance and its trend, to guide policy and decision making (Nichols & Williams, 2006; Williams et al., 2002). Beyond simply informing on the current state of the population, such demographic information can also help managers understand and predict how populations respond to stressors such as climate change (Lee et al., 2016; Wagner et al., 2023), anthropogenic disturbance (Palacios et al., 2022; Wan et al., 2022), and invasive species (Bell et al., 2021; Marschall & Crowder, 1996). For more than 60 years, ecologists and statisticians have worked to develop a suite of methods to estimate demographic parameters, abundance, and trend from capture–mark–recapture (CMR) data. While CMR‐based methods have undoubtedly provided critical information on many systems, such approaches have proven difficult to employ for certain species (Balme et al., 2009; Hupman et al., 2018; Noss et al., 1996), especially those such as boreal caribou (*Rangifer tarandus*), which inhabit remote regions, are widely dispersed, and exist at low densities.

Both methodological and technological advances, coupled with declining costs of genetic profiling, led to the rise of genetic mark–recapture methods (Luikart et al., 2010), often utilizing non‐invasive sampling techniques such as hair snares (Paetkau, 2003; Poole et al., 2001) or fecal collections (Hettinga et al., 2012; Mondol et al., 2009). While such approaches eliminate the need for the physical capture and marking of individuals, they still necessitate sampling individuals repeatedly and therefore are subject to similar investments of time, money, and personnel as traditional CMR approaches. However, recent statistical advances now allow for parameter estimation based on sampling kin pairs rather than recaptures of the same individual in a framework known as ‘close‐kin mark–recapture’ (CKMR; Bravington, Skaug, & Anderson, 2016; Skaug, 2001), thus eliminating the need for repeated sampling events.

The CKMR approach is analogous to traditional CMR, but instead of relying on recaptures, it relies on capturing closely related kin, such as parent–offspring pairs, or half‐siblings. The basic principle underlying CKMR is that the probability of sampling kin pairs, which can be assessed through their genetic profiles, is inversely proportional to the population size (Skaug, 2001). As a simple motivating example, consider a population in which all adult females give birth to the same number of offspring, on average, each year. The probability that any randomly sampled juvenile, *j*, is the offspring of a randomly sampled adult female, *f*, is simply 1/*N*_*F*_, where *N*_*F*_ is the adult female abundance alive in *j*'s year of birth. Using CKMR in most real systems requires accounting for additional complexities, such as variation in expected reproductive output, varying kinship relations, and uncertainty in kinship relations (Bravington, Skaug, & Anderson, 2016), but the basic principle remains the same.

Although CKMR has only been employed in a single terrestrial system thus far (Christmas Island flying fox [*Pteropus natalis*]; Lloyd‐Jones et al., 2023), there is growing interest in this method among terrestrial ecologists and wildlife managers (Conn et al., 2020; Larroque & Balkenhol, 2023; Sévêque et al., 2024; Sharma et al., 2022). Given that the CKMR approach does not rely on repeated capture events, it could be particularly advantageous for species which are difficult to survey via traditional CMR approaches, such as boreal caribou. Caribou are broadly distributed across much of the Canadian landscape, exhibiting large variation in ecology, genetics, behavior, and morphology (COSEWIC, 2011). In fact, caribou are considered the most widespread and variable of all Cervidae species (Geist, 1998). The Committee on the Status of Endangered Wildlife in Canada currently recognizes 12 distinct and ecologically significant ‘Designatable Units’ for caribou, although unique local herds are recognized within each Designatable Unit (COSEWIC, 2011). The local herds display immense variation in population size, with some consisting of fewer than 100 individuals, while others number over 100,000. Given the immense variation in herd size and the environs they inhabit, numerous estimation methods have been employed for different caribou herds including aerial surveys (Government of Nunavut, 2021), CMR (McFarlane et al., 2018), spatially explicit capture–recapture (SECR; McFarlane et al., 2022), and integrated population models (Moeller et al., 2021). Unfortunately, these approaches are quite costly as a result of considerable flight time through multiple survey events (CMR and SECR). Therefore, some caribou managers are interested in using CKMR to generate an abundance estimate from a one‐time sampling event. In fact, one‐time surveys have already been conducted or are otherwise planning to be conducted, in several caribou ranges, with CKMR‐based abundance estimation among the stated objectives.

To our knowledge, CKMR has only been used in cases where samples have been collected over multiple years, thereby allowing pairwise comparisons to be made across years, thus increasing the number of pairwise comparisons which result in a kinship relation and ultimately improving the precision of the resulting abundance estimate. While that is certainly the ideal scenario for generating CKMR‐based abundance estimates, unfortunately, on‐the‐ground caribou monitoring is not necessarily so consistent. Due to the large expanse over which caribou occur, and the high costs involved in their sampling, there are only so many herds that can be sampled in any given year; certain herds may be surveyed annually over the span of several years, while others may only be surveyed once every 5–10 years (depending on funding and management priorities). While it would be technically feasible to construct a multi‐year CKMR model spanning the 5–10 years between the intermittent sampling events, given that the average caribou lifespan is ~10 years, it is unlikely that there would be many mother–offspring pairs (our current focus) found between the sampling years, and therefore, a multi‐year model would offer little to no improvement over a single‐year model. Fortunately, for small populations, it may be feasible to obtain a sufficient number of kin pairs to allow for abundance estimation from a single sampling event. Here, we consider this scenario for the first time and evaluate the feasibility of using CKMR to generate an abundance estimate from just a sole, non‐invasive sampling event occurring in only a single year.

We first conduct a simulation study roughly based on caribou demographic rates to evaluate the accuracy and precision of the CKMR abundance estimate from a single, non‐invasive sampling event across a range of realistic population sizes and sampling intensities for boreal caribou. Intensive sampling of the sort of small populations considered here can result in non‐independence among the pairwise kinship comparisons, thereby impacting the variance of the abundance estimate (Bravington, Skaug, & Anderson, 2016; Skaug, 2017), and therefore, we also analyzed the coverage probability (i.e., the proportion of confidence intervals containing the true abundance across all simulations) of calculated 95% profile confidence intervals of the abundance estimate (for a single parameter, this is based on finding the two points on the likelihood surface which are 1.92 units away from the maximum value of the log‐likelihood function). We then present a case study using genetic data from previously collected fecal samples in which we use the CKMR pseudolikelihood to estimate annual adult female abundance over a 10‐year period for a small (<100), intensively sampled mountain caribou population and compare the CKMR abundance estimates to published CMR‐based abundance estimates, representing only the second use of CKMR in a real terrestrial system (Lloyd‐Jones et al., 2023).

## 2 METHODS

### 2.1 Caribou reproductive biology and fecal pellet collection

Female caribou typically give birth to their first calf as 3‐year‐olds, although a small number become pregnant at ~16 months, birthing for the first time as 2‐year‐olds (Adams & Dale, 1998a; Eloranta & Nieminen, 1986). Caribou rutting season typically runs from late September to late October, followed by a single, synchronous birth pulse in late May to mid‐June (Adams & Dale, 1998b; Dauphiné Jr & McClure, 1974). Non‐invasive fecal pellet collections are typically conducted in the winter months from late December to early March, when snow cover allows for aerial identification of cratering sites, while also helping to preserve the fecal DNA.

### 2.2 Simulation study

Briefly, we constructed individual‐based, female‐only population simulations based on realistic caribou demographic rates. We explored a range of initial population sizes (58–1150), and the simulations began immediately prior to the summer birth pulse, so 1‐year‐olds were the youngest individuals at the start of each simulation. We then simulated the birth of a single calf cohort, using stage‐specific breeding probabilities, and tracked which individuals gave birth to which calves. Given that caribou sampling generally occurs in the winter (6–8 months after the births), we simulated a 6‐month survival process for all individuals, again using stage‐specific survival probabilities. Following the survival process, we randomly sampled between 5% and 95% of the surviving population, determined how many mother–calf pairs were contained within the sample, and used this information from the one‐time sample to estimate the reproductive female abundance at the time of the calf cohort's birth. Below, we further elaborate on the details of the simulations.

The starting population for each simulation consisted of three stage‐classes: yearlings (1‐year‐olds), which are non‐reproductive, subadults (2‐year‐olds), and adults (3+‐year‐olds), both of which are reproductive; no calves (age 0) were present at the start of the simulations. The reproductive portion of each population (subadults and adults; *N*_*F*_) ranged in size from 50 to 1000 females, in increments of 50, with subadults accounting for 12% of the reproductive individuals and adults accounting for the remaining 88%, while the number of yearlings was set at 15% of the total number of reproductive individuals. Therefore, total initial population sizes ranged from 58 to 1150 females.

Next, we used a Bernoulli distribution to determine whether each female successfully bred, with the breeding probability determined by each individual's stage‐class (see Table 1 for the full list of demographic rates used in the simulations). Females that bred successfully produced one calf, regardless of stage‐class, consistent with caribou demography (Bergerud, 2000), and we tracked which female produced each calf within the simulation. Given that caribou sampling often occurs in the winter months (6–8 months after the summer birth pulse), we then used a Bernoulli distribution to simulate the survival process for ~6 months for all stage‐classes, where the survival probability was determined by each individual's stage‐class. We then randomly sampled 5%–95% of all surviving individuals, in 5% increments, for each population to assess the impact of sampling differing proportions of individuals on the abundance estimate.

**TABLE 1:** Demographic parameters used to generate the population subjected to sampling within each simulation.

Parameter	Symbol	Value
Numb of offspring born per female which reproduced	*f*	1
Yearling breeding probability^a^	—	0
Subadult breeding probability	*b*_*s*_	.15
Adult breeding probability	*b*_*a*_	.90
Calf 6‐month survival	$\phi_{c}$	.35
Yearling 6‐month survival	$\phi_{y}$	.894
Subadult 6‐month survival	$\phi_{s}$	.894
Adult 6‐month survival	$\phi_{a}$	.922

^a^ This parameter is not actually part of the simulations because the probability is 0, but we include here for completeness.

Following sampling, all sampled yearlings were removed from consideration for the subsequent CKMR analysis because they are neither calves (hence not part of the offspring cohort of interest), nor are they capable of reproducing yet (hence having no chance of being a mother to the current calf cohort). As a result, all individuals included in the CKMR analysis as potential mothers were known to be reproductive at the time of the calf cohort's birth. We then made all possible pairwise comparisons between the sampled calves and reproductive females, determined how many mother–calf pairs were sampled, and used the mother–offspring kinship probability (Bravington, Skaug, & Anderson, 2016) to estimate the number of reproductive females at the time of the calf cohort's birth. In this approach, each candidate mother belonged to one of two stage‐classes (subadult or adult), and each pairwise comparison either yielded a mother–calf pair, or not. Therefore, each pairwise comparison could be placed into one of four categories: (1) a comparison involving a subadult, which was a mother–calf pair; (2) a comparison involving a subadult which was not a mother–calf pair; (3) a comparison involving an adult, which was a mother–calf pair; and (4) a comparison involving an adult which was not a mother–calf pair. Thus, the pseudolikelihood of the observed data can be expressed simply using a multinomial:

Lpn,y=nyip1y1p2y2p3y3p4y4,

where ***p*** corresponds to a vector containing the probabilities of each of the four possible outcomes for a given pairwise comparison, ***y*** corresponds to a vector containing the frequency of each of the four outcomes, and *n* corresponds to the total number of pairwise comparisons made (i.e., the sum of ***y***). The equation governing the probability of each outcome is displayed in Table 2.

**TABLE 2:** The probability of the possible outcomes from each pairwise comparison as used in the multinomial likelihood.

Outcome	Probability
Subadult comparison yields a mother–calf pair	$\frac{b_{s}f}{N_{F}\left(b_{s}f\left(1-A\right)+b_{a}\mathrm{fA}\right)}$
Subadult comparison does not yield a mother–calf pair	$1-\frac{b_{s}f}{N_{F}\left(b_{s}f\left(1-A\right)+b_{a}\mathrm{fA}\right)}$
Adult comparison yields a mother–calf pair	$\frac{b_{a}f}{N_{F}\left(b_{s}f\left(1-A\right)+b_{a}\mathrm{fA}\right)}$
Adult comparison does not yield a mother–calf pair	$1-\frac{b_{a}f}{N_{F}\left(b_{s}f\left(1-A\right)+b_{a}\mathrm{fA}\right)}$

*Note*: *A* represents the proportion of adults among the sampled reproductive (subadults and adults) females; all other symbols are as defined in Table 1. The denominator of the fraction represents the total expected reproductive output across all potentially reproductive females.

We used numerical maximization to find the maximum (pseudo)likelihood estimate of $\hat{N_{F}}$, searching across the range of 10 females to 10 times the number of reproductive females. Although the pseudolikelihood is not a proper likelihood function, when the necessary assumptions are met and the pseudolikelihood approximates a proper likelihood, the 95% confidence interval of $\hat{N_{F}}$ can be approximated using Hessian‐based confidence intervals or other standard approaches. However, it is unclear how well these approximations perform when the pseudolikelihood may not approximate a proper likelihood due to violations of one or more of its underlying assumptions, as is the case under consideration here. Therefore, we were also interested in assessing the coverage probability of the calculated 95% confidence interval, so we computed the 95% profile confidence interval of $\hat{N_{F}}$ for each simulation. We performed 10,000 simulations for each considered population size and computed the mean $\hat{N_{F}}$ across all simulations at each sampling intensity, as well as the 95% quantile for $\hat{N_{F}}$, the standard deviation, the coefficient of variation, and the proportional relative bias ($\hat{(N_{F}}-N_{F})/N_{F}$). All simulations and analyses were performed in the R computing environment version 4.0.5 (R Core Team, 2016).

### 2.3 Case study: Tonquin herd, Alberta, Canada

#### 2.3.1 Sample collection, DNA analysis, and age class determination

Our Tonquin case study utilized data presented in previous publications (Flasko et al., 2017; McFarlane et al., 2018). Briefly, fecal pellets were collected from the Tonquin subpopulation in Jasper National Park, Alberta, Canada, each winter from 2006 to 2015, with two or three fecal surveys conducted annually between October and January. Following DNA extraction, samples were amplified at 15 variable microsatellite loci (McFarlane et al., 2018, 2021, 2022), and unique individuals were identified using the ALLELEMATCH package (Galpern et al., 2012) in R. Fecal pellets from the unique individuals were then assigned to either calf or non‐calf (which consists of yearling, subadult, and adult) stage‐classes in their first year of capture based on a combination of fecal pellet dry‐weight and hormone levels (pregnane for females, testosterone for males). For a full description of the age‐class determination methods, please see Flasko et al. (2017) and McFarlane et al. (2018). Because the birth year was known for all individuals initially captured as calves, their ages were known on any encounters in subsequent years.

#### 2.3.2 Mother–offspring pair inference

We used COLONY v2.0.6.8 (Jones & Wang, 2010), which uses a maximum likelihood framework, to infer parent–offspring relationships among the sampled calves and non‐calves. All sampled calves were input as offspring. Because individuals sampled as calves could be the parent of a calf in future years, all sampled females, both calves and non‐calves, were included in COLONY as candidate mothers. Although we were not directly interested in paternity, all sampled males were included as candidate fathers to enhance the quality of parent‐pair (and thus maternity) inference. COLONY also allows the exclusion of certain candidate mothers (or fathers) from consideration for maternity (or paternity) of specific offspring based on prior information, such as age. Therefore, because the age of first reproduction is generally 3 years old for both sexes (Adams & Dale, 1998a, 1998b; Eloranta & Nieminen, 1986), all individuals which were known to be younger than 3 years old (owing to having been captured as a calf in a previous year) at the time of a given calf's birth were excluded from parental consideration for said calf. See Table A1 in Appendix for the full set of COLONY input parameters.

#### 2.3.3 Abundance estimation

Due to sample size concerns, we pooled together data from all sampling occasions which occurred within the same winter (2–3 occasions per winter), and treated it as arising from a single, intensive sampling event. Although the Jasper dataset spans multiple years, given caribou managers' interest in using CKMR in a one‐time‐only sample design, we analyzed the data on a year‐by‐year basis, forgoing all cross‐year comparisons, as they would not be available should a one‐time sampling design be implemented. Therefore, we only included individuals who were sampled in the same year in our analysis. Within a given year, we counted the number of sampled calves, the number of candidate mothers who were sampled in the year of interest and were not excluded due to being younger than 3‐year‐olds (hereafter referred to as ‘potential mothers’), and the number of mother–calf pairs sampled within said year as indicated by COLONY. Upon reaching sexual maturity, female caribou are expected to produce one calf annually (Bergerud, 2000). Given this constant fecundity in combination with the fact that we only evaluate kinship comparisons between potential mothers and calves‐of‐the‐year, the expected relative reproductive output for any given potential mother is assumed to be 1/*N*_*f*_, where *N*_*f*_ represents the reproductive female abundance in the offspring's year of birth. Therefore, the number of mother–calf pairs (MO_*t*_) found within a given year *t* can be succinctly expressed as

$$MO_{t}\sim \text{Binom}\left(n_{c,t}*n_{f,t},\frac{1}{N_{f,t}}\right),$$

where *n*_*c,t*_ and *n*_*f,t*_ represent the number of sampled calves and the number of sampled potential mothers in year *t*, respectively, and *N*_*f,t*_ is the reproductive female abundance in year *t*. Notably, this formulation treats all potential mothers as reproductive adults, which is not strictly true because the potential mothers also include some yearlings (non‐reproductive) and subadults (occasionally reproductive), which could not be excluded due to having an unknown birth year. Although this inevitably introduces some degree of bias into our abundance estimates, we nevertheless believe that this formulation is a reasonable approximation (See Section 4). We used the ‘binom.confint’ function from the ‘binom’ package in R (Dorai‐Raj, 2022) to estimate *N*_*f,t*_ as well as its 95% profile confidence interval for each year.

## 3 RESULTS

### 3.1 Simulation study

Our simulation study used the CKMR pseudolikelihood to evaluate the accuracy and precision of the CKMR abundance estimator from a single sampling event. Although we simulated reproductive female populations ranging in size from 50 to 1000 in increments of 50, in what follows we only present the results for six population sizes (50, 100, 250, 500, 750, and 1000), which are representative of the patterns found across all simulations. The mean number of observed mother–offspring pairs increased as both the population size and the proportion of the population sampled increased (Table 3). While the mean number of mother–offspring pairs was extremely low (<1.0) when only 5% of the population was sampled, regardless of population size, the disparity in the number of observed mother–offspring pairs among the population sizes grew as the proportion of the population sampled increased. For example, with only 25% of the population sampled, the population sizes of 250 (sample size: *n* = 63) and 1000 (*n* = 250) had a mean of 4.1 and 16.3 observed mother–offspring pairs, respectively; these grew to 36.7 and 146.9, respectively, when 75% (*n* = 188 and *n* = 750, respectively) of the population was sampled (Table 3).

**TABLE 3:** The mean number of mother–offspring pairs (MOPs) across all 10,000 simulations for each combination of population size and the proportion of the population sampled.

Mean number of MOPs																			
Pop. size	Proportion sampled																		
	0.05	0.10	0.15	0.20	0.25	0.30	0.35	0.40	0.45	0.50	0.55	0.60	0.65	0.70	0.75	0.80	0.85	0.90	0.95
50	0.0	0.1	0.3	0.5	0.8	1.1	1.6	2.0	2.6	3.2	3.9	4.7	5.5	6.3	7.4*	**8.3**	**9.4**	**10.5**	**11.7**
100	0.1	0.2	0.6	1.0	1.6	2.3	3.2	4.1	5.3	6.5	7.9	9.3*	11.1*	**12.8**	**14.7**	**16.7**	**18.9**	**21.1**	**23.5**
250	0.2	0.6	1.5	2.6	4.1	5.9	7.9	10.4*	13.2*	**16.3**	**19.7**	**23.4**	**27.6**	**32.0**	**36.7**	**41.7**	**47.1**	**52.9**	**59.0**
500	0.3	1.3	2.9	5.2	8.1	11.7*	15.9*	**20.9**	**26.5**	**32.7**	**39.5**	**46.9**	**55.2**	**64.0**	**73.6**	**83.6**	**94.4**	**105.8**	**117.9**
750	0.5	2.0	4.4	7.8	12.2*	17.6*	**24.0**	**31.3**	**39.7**	**49.0**	**59.4**	**70.6**	**82.8**	**96.2**	**110.4**	**125.6**	**141.8**	**158.9**	**177.1**
1000	0.7	2.6	5.9	10.4	16.3*	**23.4**	**32.0**	**41.7**	**52.9**	**65.2**	**78.8**	**93.9**	**110.2**	**127.8**	**146.9**	**167.1**	**188.5**	**211.5**	**235.6**

*Note*: Bolded values represent combinations of population size and proportion of the population surveyed which yielded a proportional relative bias ≤0.1 and a CV ≤0.2, while those followed by a ‘*’ denote combinations with a proportional relative bias ≤0.1 and a CV between 0.2 and 0.3.

**FIGURE 1:**
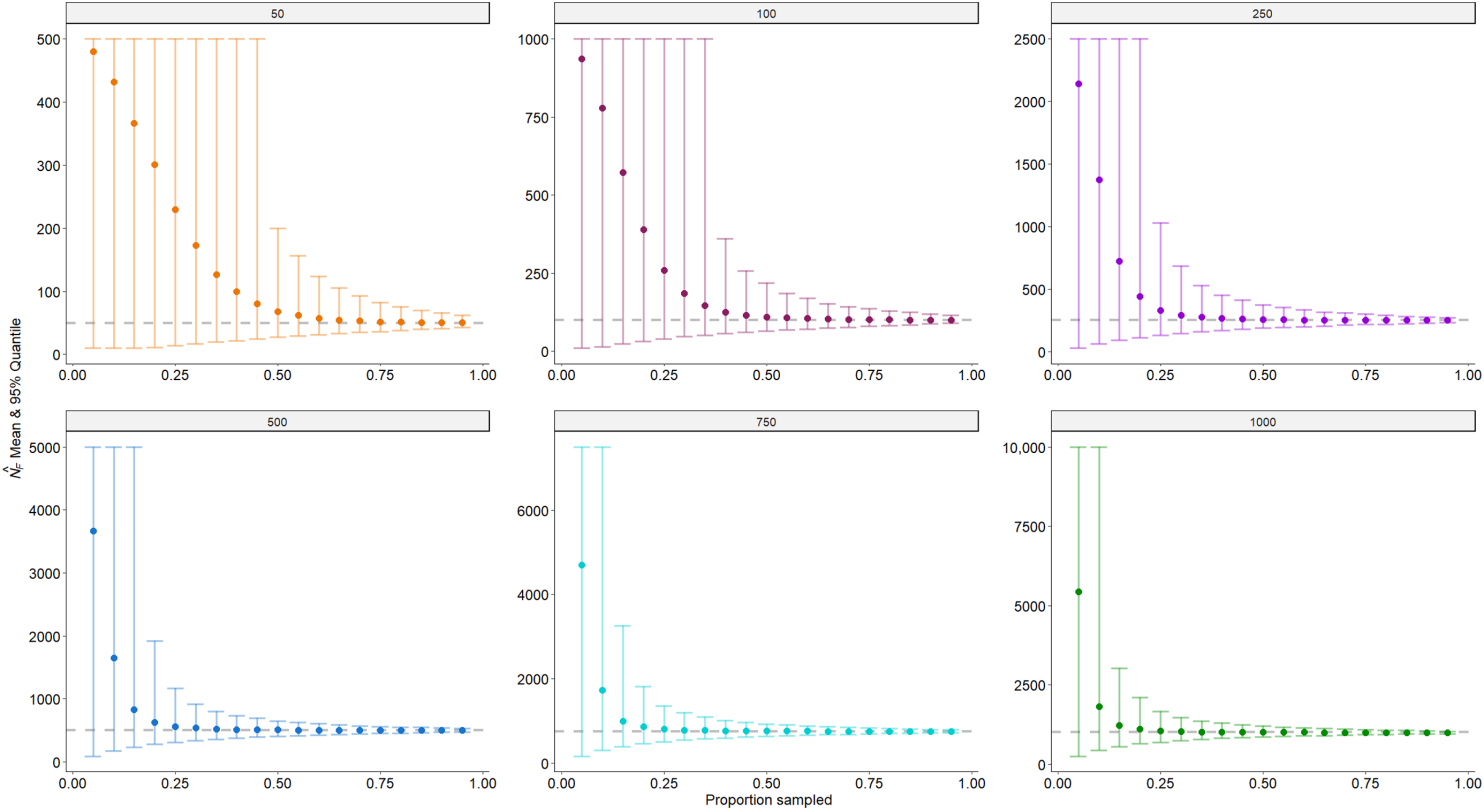
The mean (colored dots) and 95% quantile (colored bars) of the CKMR abundance estimate across all 10,000 simulations for each proportion of the population sampled for selected population sizes. In each plot, the dashed gray line represents the true reproductive female abundance.

As may be expected, our simulations indicated that both the accuracy and precision of the abundance estimator improved with both increasing population size and the proportion of the population sampled (Figure 1). Regardless of the population size, when very small proportions (≤10%) of the population were sampled, the mean abundance estimate was biased high with very high uncertainty; the corresponding mean relative bias was also quite large, ranging from 0.82 to 8.60 (Table 4). As the proportion of the population sampled increased, the mean abundance estimate converged to the true value, the mean proportional relative bias declined to 0, and the precision of the estimator improved (Figure 1; Table 4). While the mean abundance estimates eventually converged to the true value for all population sizes, smaller populations required a larger proportion of the population to be sampled before convergence occurred. For example, the population size of 50 required sampling ~65% (*n*≈33) of the population before the mean proportional relative bias was ≤0.1, indicating the abundance estimate was within 10% of the true value, while the population size of 1000 only required sampling ~20% (*n*≈200) of the population before achieving the same level of accuracy (Table 4; Figure 1). Interestingly, the median proportional relative bias declined faster than the mean proportional relative bias as larger proportions of the population were sampled, especially for smaller populations. In fact, achieving a median proportional relative bias ≤0.1 only required sampling ≤25% of the population across all considered population sizes (Table 4). This behavior can be explained by the fact that CKMR vastly overestimates the population size in cases where very few (or only one) mother–offspring pairs are found, which occurs more often when sampling small proportions of small populations, thus inflating the mean relative bias while having a much smaller impact on the median relative bias.

**TABLE 4:** The mean and median proportional relative bias ($\left(\hat{N_{F}}-N_{F}\right)/N_{F}$) across all 10,000 simulations for each combination of population size and the proportion of the population sampled.

Proportional relative bias																				
Pop. size	Metric	Proportion sampled																		
		0.05	0.10	0.15	0.20	0.25	0.30	0.35	0.40	0.45	0.50	0.55	0.60	0.65	0.70	0.75	0.80	0.85	0.90	0.95
50	Mean	8.60	7.64	6.33	5.02	3.59	2.46	1.53	1.00	0.60	0.36	0.23	0.14	0.09	0.07	0.04	0.03	0.02	0.01	0.00
	Median	9.00	9.00	9.00	9.00	0.04	0.04	0.02	0.02	0.00	0.00	0.00	−0.02	0.00	0.00	−0.02	−0.02	−0.02	0.00	0.00
100	Mean	8.36	6.79	4.73	2.89	1.59	0.85	0.47	0.25	0.15	0.10	0.07	0.05	0.03	0.02	0.02	0.01	0.01	0.00	0.00
	Median	9.00	9.00	9.00	0.08	0.04	0.03	0.02	0.01	0.00	−0.01	0.00	0.00	−0.01	−0.01	0.00	0.00	−0.01	0.00	0.00
250	Mean	7.57	4.49	1.89	0.77	0.33	0.17	0.10	0.06	0.4	0.02	0.02	0.02	0.01	0.01	0.00	0.00	0.00	0.00	0.00
	Median	9.00	9.00	0.12	0.03	0.01	0.00	0.00	0.00	0.00	−0.01	−0.01	0.00	0.00	0.00	0.00	0.00	0.00	0.00	0.00
500	Mean	6.33	2.29	0.67	0.25	0.12	0.07	0.04	0.03	0.02	0.01	0.01	0.01	0.00	0.00	0.00	0.00	0.00	0.00	0.00
	Median	9.00	0.18	0.04	0.02	0.01	0.00	0.00	0.00	−0.01	0.00	0.00	0.00	0.00	0.00	0.00	0.00	0.00	0.00	0.00
750	Mean	5.26	1.30	0.32	0.14	.07	0.04	0.02	0.02	0.01	0.01	0.00	0.00	0.00	0.00	0.00	0.00	0.00	0.00	0.00
	Median	9.00	0.03	0.02	0.01	0.01	0.00	0.00	0.00	0.00	0.00	−0.01	0.00	0.00	0.00	0.00	0.00	0.00	0.00	0.00
1000	Mean	4.43	0.82	0.22	0.10	0.04	0.03	0.02	0.01	0.01	0.00	0.00	0.00	0.00	0.00	0.00	0.00	0.00	0.00	0.00
	Median	9.00	0.05	0.02	0.01	0.01	0.00	0.00	0.00	0.00	0.00	0.00	0.00	0.00	0.00	0.00	0.00	0.00	0.00	0.00

**FIGURE 2:**
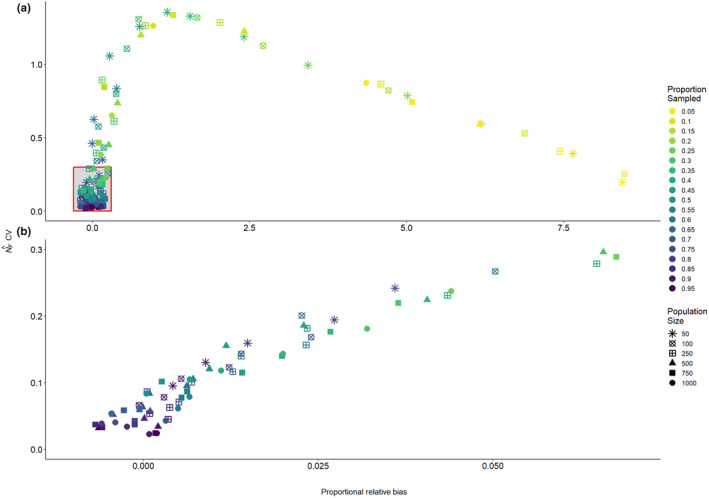
(a) Proportional relative bias $\left(\left(\hat{N_{F}}-N_{F}\right)/N_{F}\right)$ versus the coefficient of variation of $\hat{N_{F}}$ for each combination of population size and proportion of the population sampled. The red box in the lower‐left region of the plot represents combinations with both a low relative bias and a relatively low (≤0.30) coefficient of variation, such that the estimates could be both accurate and precise enough for management purposes. (b) Zoomed‐in view of the region of the graph highlighted by the red box in (a).

To evaluate both the accuracy and the precision of the abundance estimator simultaneously, we plotted the mean proportional relative bias of the abundance estimate against its coefficient of variation for every combination of population size and proportion of the population sampled (Figure 2a). Again, we see that low sampling proportions lead to high proportional relative bias regardless of the population size. However, the points in the lower‐left region of the graph, contained within the red box, are of particular interest, as these represent combinations of population size and proportion of the population sampled, which yield an estimate with a mean proportional relative bias of ≤0.1 (i.e., within 10% of the true value) and a coefficient of variation of ≤0.30. Although a CV ≤0.20 is typically desired, there may be some circumstances in which management is willing to accept a slightly higher CV, hence why we used the threshold of 0.30 here. There is a range of combinations which yield points within this region of the graph (Figure 2b). For example, a population size of 50 requires ≥75% (*n* ≥ 38) of the population to be sampled to yield a mean estimate that is both accurate and precise enough to fall within this region of the graph, a population size of 250 requires ≥40% (*n* ≥ 100) of the population to be sampled, while a population size of 1000 only requires ≥20% (*n* ≥ 200) of the population to be sampled (Figure 2b).

**FIGURE 3:**
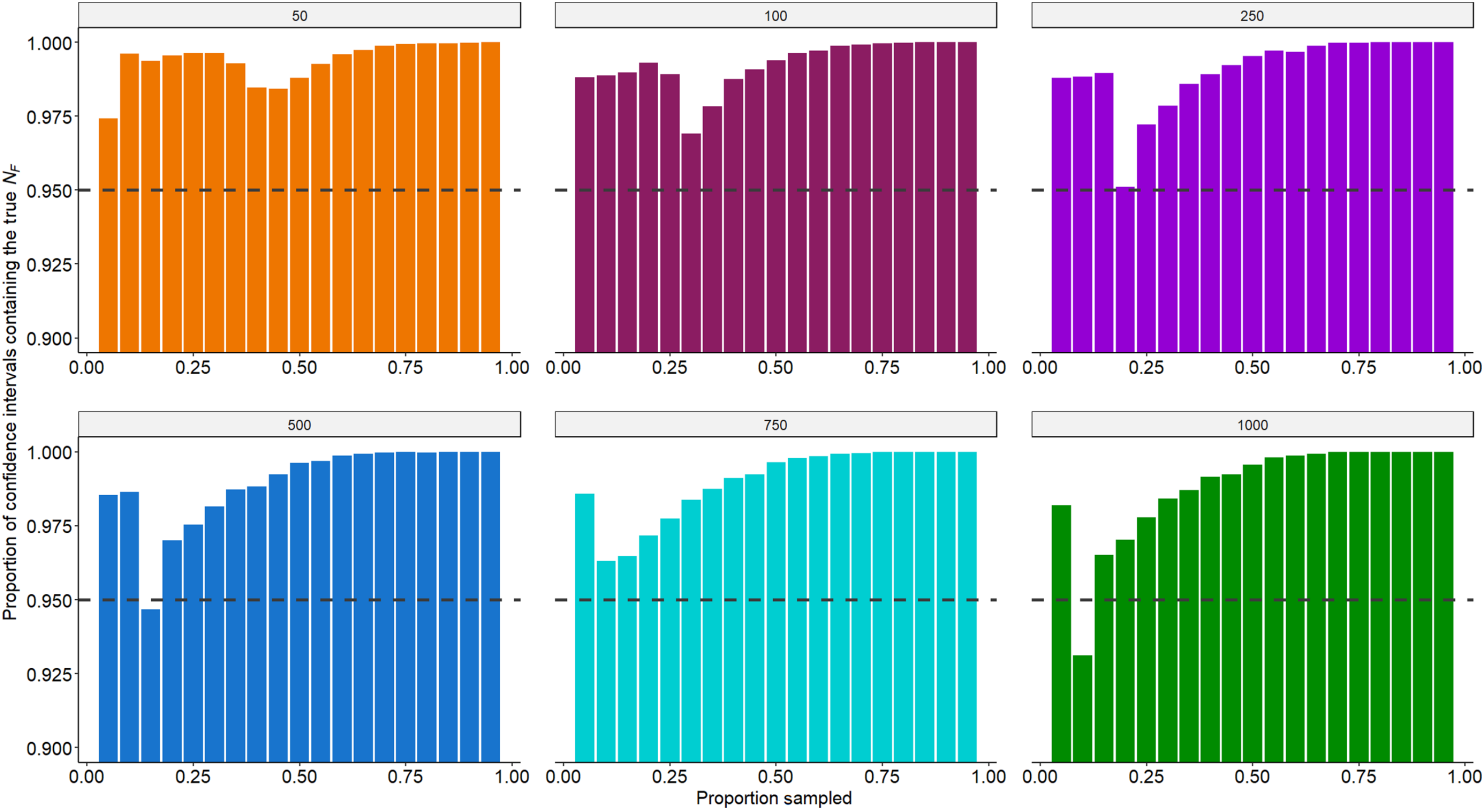
The proportion of the calculated 95% profile confidence intervals which contain the true abundance across all 10,000 simulations for each combination of population size and proportion of the population sampled.

Lastly, we found that the calculated 95% profile confidence interval computed in each simulation tended to be overly broad, thereby misrepresenting the actual coverage probability. In fact, for many combinations of population size and proportion of the population sampled, the proportion of the 95% profile confidence intervals containing the true *N*_*F*_ across all 10,000 simulations was ≥99%, even reaching 100% in some cases (Figure 3).

### 3.2 Case study: Tonquin herd, Alberta, Canada

**FIGURE 4:**
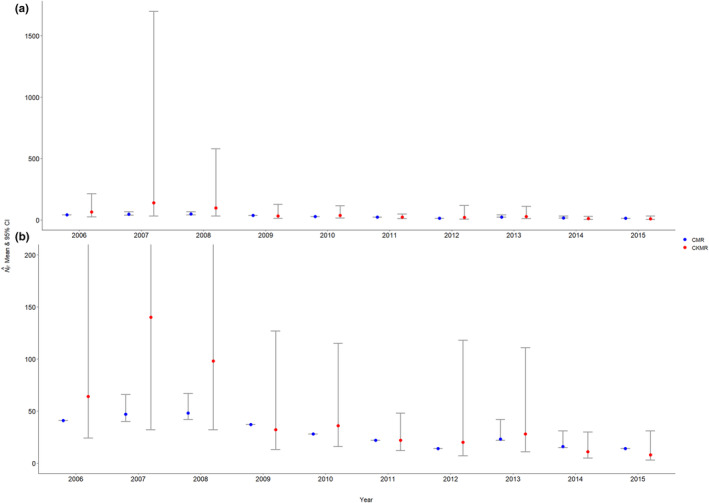
(a) Annual CMR (blue) and CKMR (red) female abundance estimates and their associated 95% confidence intervals (gray bars) for Jasper National Park, Alberta, Canada. The CMR results and their associated 95% confidence intervals are reported in tab. A7 of McFarlane et al. (2018). (b) Zoomed‐in view of (a).

The number of calves sampled within each year ranged from a minimum of 3 in 2015 to a maximum of 11 in 2011, and the number of potential mothers sampled ranged from a minimum of 8 in 2015 to a maximum of 32 in 2006 and 2009, while the number of mother–calf pairs found among the sampled individuals ranged from 1 in 2007 to 8 in 2011 (Table 5). The performance of the CKMR female abundance estimate varied across years. The estimates were both inaccurate and imprecise for 2006–2008 compared to existing robust‐design CMR female abundance estimates previously published in McFarlane et al. (2018), with especially poor performance in 2007 and 2008 (Figure 4a). The CKMR‐based abundance estimates were more reasonable and comparable to the CMR estimates for 2009–2015 and the precision of the estimates also improved during this period (compared to the first 3 years; Figure 4a), although were still relatively imprecise in certain years given the low abundance estimates (Figure 4b). For example, the 95% profile confidence interval spanned 13–127 in 2009 ($\hat{N_{F}}$ = 32) and 7–118 in 2012 ($\hat{N_{F}}$ = 20). The mean CMR‐based female abundance estimate across the 10‐year study period was 29, while our mean CKMR‐based estimate was 45.9 (Table 5). However, after excluding the first 3 years during which CKMR performed particularly poorly, the 7‐year mean CMR‐based female abundance estimate was 22, while the 7‐year mean CKMR‐based estimate was 22.43, highlighting the improved performance after the first 3 years.

**TABLE 5:** Summary information for the annual surveys in Jasper National Park, Alberta, Canada.

Year	No. of calves (both sexes) sampled	No. of potential mothers sampled	No. of pairwise comparisons	No. of MCPs found	% pairwise comparisons yielding MCP	No. of non‐calf females excluded from potential mothers	Total No. of individuals sampled	CKMR *N*_*F*_ (95% CI)	CMR *N*_*F*_ (95% CI)	CMR N_Total_ (95% CI)	Estimated % population sampled
2006	8	32	256	4	1.56%	0	61	64 (24, 215)	41 (41, 41)	65 (65, 65)	93.85%
2007	5	28	140	1	0.71%	2	50	140 (32, 1699)	47 (40, 66)	78 (58, 98)	64.10%
2008	7	28	196	2	1.02%	5	67	98 (32, 580)	48 (42, 67)	87 (72, 102)	77.01%
2009	3	32	96	3	3.13%	3	63	32 (13, 127)	37 (37, 37)	74 (65, 83)	85.14%
2010	6	24	144	4	2.78%	1	54	36 (16, 115)	28 (28, 28)	55 (55, 55)	98.18%
2011	11	16	176	8	4.55%	2	46	22 (12, 48)	22 (22, 22)	50 (44, 56)	92.00%
2012	4	10	40	2	5%	2	27	20 (7, 118)	14 (14, 14)	37 (26, 48)	72.97%
2013	7	12	84	3	3.57%	2	28	28 (11, 111)	23 (22, 42)	53 (33, 74)	52.83%
2014	7	8	56	5	8.93%	1	26	11 (5, 30)	16 (15, 31)	35 (29, 41)	74.29%
2015	3	8	24	3	12.5%	0	19	8 (3, 31)	14 (14, 14)	26 (26, 26)	73.08%
Avg.	6.1	20	122.8	3.5	4.37%	1.8	44.1	45.9	29	56	78.35%

*Note*: ‘MCP’ means ‘mother–calf pair.’ The number of ‘non‐calf females excluded from potential mothers’ corresponds to the number of non‐calf females who were known to be under 3 years old (due to being a known‐age individual as a result of being previously captured as a calf). The ‘CKMR *N*_*F*_’ is the estimated reproductive female abundance from our CKMR analysis, while the ‘CMR *N*_*F*_’ is the estimated total female abundance (inclusive of all stage‐classes) as reported in tab. A7 of McFarlane et al. (2018); likewise, the ‘CMR *N*_Total_’ is the estimated total abundance (inclusive of both sexes) as reported in tab. A6 of McFarlane et al. (2018). The number of individuals sampled reported here differs slightly from those reported in tab. A2 of McFarlane et al. (2018) due to updated quality control procedures.

## 4 DISCUSSION

Unlike traditional CMR methods, the CKMR approach does not rely on repeated capture events, which, in principle, means it could be possible to generate an abundance estimate from a single sampling event. Caribou managers are particularly interested in this possibility. However, applications of CKMR thus far have focused on systems in which samples have been collected over a span of multiple years, allowing pairwise comparisons to be made across years, thus increasing the number of kin pairs included in the analysis and ultimately improving the precision of the resulting abundance estimate. Fortunately, small populations have the advantage that it may be feasible to collect enough kin pairs for CKMR from a single sampling event, something which is generally infeasible for larger populations. To our knowledge, our study is the first to assess the suitability of CKMR for small populations using samples collected from a single, non‐invasive sampling event, while also representing only the second manuscript to use CKMR in a real terrestrial system (Lloyd‐Jones et al., 2023).

Our simulations demonstrate that the CKMR mother–offspring approach can, in principle, generate estimates of reproductive female abundance that are accurate and precise enough to be useful for caribou management (i.e., CV ≤ 20%, Pollock et al., 1990) from a single, non‐invasive sampling event, given sufficient sampling intensity. However, the sampling intensity required to achieve an abundance estimate with both a sufficiently low proportional relative bias and CV depends on the population size (Figure 2). Ideally, this means that managers should have some idea of their population size prior to conducting a survey in order to determine how many samples are likely to be required to achieve the desired level of accuracy and precision. Across our simulations, the 95% profile confidence intervals for $\hat{N_{F}}$ tended to be overly broad, likely because the pseudolikelihood underlying the CKMR approach may not adequately approximate a proper likelihood function when the assumption of approximate independence among the pairwise comparisons is violated, highlighting the need for caution when reporting or otherwise interpreting confidence intervals for CKMR‐based abundance estimates for small, intensively sampled populations.

The accuracy and precision of the annual CKMR‐based abundance estimates varied across years for our Tonquin, Alberta, caribou case study. The abundance estimates were biased high with large uncertainty for 2006–2008 but performed reasonably in subsequent years. Notably, during each of the first 3 years, the percentage of pairwise comparisons between calves and potential mothers which resulted in a mother–calf pair was <1.5%, while in all subsequent years, it was >2.5%, even reaching ≥5% in several of the later years of the study period (Table 5), highlighting the importance of sampling a sufficient number of kin pairs. It has previously been suggested that CKMR studies should generally target obtaining a minimum of 50 kin pairs (Bravington, Skaug, & Anderson, 2016; Waples & Feutry, 2021). Of course, obtaining 50 mother–calf pairs from a single sampling event can be impossible in certain scenarios depending on the population size, as evidenced by both our simulations and case study. While our results indicate that reasonable abundance estimates can be obtained from fewer than 50 mother–calf pairs for small, intensively sampled populations given sufficient sampling intensity, precision of the estimator, as measured by the width of the 95% confidence interval, is certainly improved with increasing numbers of mother–calf pairs, and therefore, targeting ~50 kin pairs is still a useful heuristic where logistically feasible. Additionally, although we report the 95% profile confidence interval for the abundance estimates from our Jasper case study, our simulations strongly suggest that the coverage of this confidence interval is overly broad and should therefore be viewed with some caution.

While the CKMR‐based abundance estimates are similar to the CMR‐based estimates from McFarlane et al. (2018) from 2009 onward, there are several details which should be noted. First, our analysis assumed that all potential mothers have identical expected reproductive output. This is not strictly true because we could not distinguish between yearlings, subadults, and adults, except in cases where an individual was previously captured as a calf and therefore its age was known. The presence of non‐reproductive individuals within the set of potential mothers in any given year would artificially increase the number of pairwise comparisons yielding a non‐kin relationship, while having no impact on the number of comparisons yielding a mother–offspring pair, thereby decreasing the ratio of kin/non‐kin comparisons and ultimately causing the abundance estimate to overestimate the reproductive female abundance to some degree. However, given the relatively high estimated proportion of the population sampled in each year (average of ~78% annually), we are reasonably confident that the majority of calves were sampled in any given year, thus representing samples of known age on encounters in subsequent years (and able to be excluded from the potential mothers until they turned 3 years old). Furthermore, we expect only a relatively small proportion of the population in any given year to consist of yearlings; therefore, we expect the number of individuals captured for the first time as yearlings, and thus incorrectly included in the group of potential mothers in any given year, to be small. Nevertheless, future studies could circumvent the difficulties that we encountered in stage‐class assignment through the development of a caribou‐specific epigenetic clock (Czajka et al., 2024; Lu et al., 2023), which would then allow for the estimation of each individual's true age. Obtaining the true age of each individual would be particularly advantageous because it would enable managers to retrospectively estimate the abundance for multiple years from just a single sampling event instead of only being able to estimate the abundance for the mothers of the current calf cohort, as we have done throughout this study. Additionally, age information could also be useful for other management questions, such as those related to individual fitness levels.

Second, CKMR and CMR estimate similar, although slightly different parameters. The CMR‐based estimates from McFarlane et al. (2018) represent the total number of females (regardless of age), which were physically present at the time of sampling, while the CKMR‐based estimates presented here represent the number of reproductive females who were present at the time of a given calf cohort's birth, approximately 6 months prior to sampling. Because our CKMR‐based estimates do not include non‐reproductive females, while the CMR‐based estimates do, we would expect our CKMR‐based estimates to be smaller than the CMR‐based estimates, which are inclusive of all stage‐classes. Therefore, while the mean CMR‐based abundance estimate for 2009–2015 and our mean CKMR‐based estimate for the same timeframe are virtually identical (22 and 22.43, respectively), this suggests that the CKMR‐based estimates overestimate the reproductive female abundance to some degree. As described previously, this is exactly what we would expect to observe if some non‐reproductive individuals were incorrectly included in the set of potential mothers. While we maintain that this was not a common occurrence within this dataset for the reasons described previously, it is a likely indication that it did occur in some instances. Additionally, the precision of the two abundance estimation methods is not perfectly comparable. It is likely that the model from McFarlane et al. (2018) suffered from convergence or parameter identifiability issues given that their abundance estimates had a standard error of zero in several years, which is unlikely to be an accurate assessment of the true uncertainty of their estimates; meanwhile, our confidence intervals are likely overly broad, as noted previously.

Lastly, each of our abundance estimates were generated using only a single year's data. In contrast, the abundance estimates from McFarlane et al. (2018) were generated by a robust‐design CMR model, which allowed information within the dataset to be shared across years. Of course, it is unsurprising that a model that allows information to be shared across years has greater precision than a model making use of only a single year's data at a time. It is possible to build CKMR models which make use of data collected across multiple years, and this has been the focus of the CKMR literature thus far. And, when multiple years of data are available (such as for our Jasper dataset), that is precisely what should be done for realistic management scenarios. However, given that some caribou managers are interested in attempting CKMR from a single sampling event we opted to only make use of a single year's data at a time, forgoing all cross‐year comparisons.

Our simulations and case study demonstrate the suitability of CKMR for application to small caribou populations using samples collected from a single, non‐invasive sampling event. While our results indicate that it should be achievable in practice (subject to sufficient sampling intensity), we urge caution in generalizing our results across systems. First, our simulations assumed perfect reconstruction of mother–calf pairs and that the stage‐class of every sampled individual was known perfectly. However, in real systems there will be uncertainties and errors in the assignment of kinship relations from genetic data, the severity of which will depend upon the type and number of markers used, the quality and quantity of the genetic samples, as well as the background relatedness of individuals in the population (Csilléry et al., 2006; Foroughirad et al., 2019; Konovalov & Heg, 2008; Milligan, 2003; Van Horn et al., 2008); there may also be uncertainty and errors in the assignment of stage‐class for certain individuals (as is likely the case for some individuals, particularly yearlings, in our Jasper caribou case study). These uncertainties and errors will impact the performance of the abundance estimator. Additionally, our simulations only considered the female portion of the population, with no consideration of males. While this should be sufficient for the purposes of caribou management, the particularities of other systems, as well other management interests, could necessitate the consideration of the male portion of the population. Finally, our simulation did not allow for movement of individuals into or out of the study area. As with conventional CMR, the movement of individuals could result in a biased estimate from CKMR, for example, in situations where processes such as natal dispersal are large relative to the sampling area, such that many of the offspring produced by the adults within the sampling area are not available for sampling. While we do not expect this to be an issue for boreal caribou given the large expanses over which sampling typically occurs, it could be more problematic for other species. For these reasons, we recommend that researchers and managers conduct a simulation study using their target species' life history and behavior, as well as the anticipated data uncertainties, prior to attempting a single‐sample CKMR analysis. Our simulation code could serve as a starting point for such an analysis.

Close‐kin mark–recapture‐based estimation methods are still quite new, and thus far, much of the work in this field has focused on aquatic systems, although several recent papers have explored CKMR in the context of terrestrial systems (Conn et al., 2020, Sharma et al., 2022, Larroque & Balkenhol, 2023, Lloyd‐Jones et al., 2023, Sévêque et al., 2024). However, there is still a need for more work on CKMR survey design, especially for terrestrial species. For example, given that the CKMR framework relies on sampling a sufficient number of kin pairs, randomly sampling the population of interest may not be the optimal approach and alternative sampling schemes designed to target specific cohorts/life stages at different times of the year or locations may be required (Bravington, Grewe, & Davies, 2016); if a more targeted survey design is implemented, it is still important that the collected samples be random with respect to kinship. Furthermore, the field would benefit from investigations into the effects of non‐independence of kin pairs which may arise among mother and offspring prior to dispersal (Jones et al., 2023), or among other kin pairs in group‐living species.

One of the attractive features of CKMR‐based estimation methods is that in principle, they can eliminate the need for multiple sampling events as is typically required by CMR or SECR approaches, thus representing a potential cost savings to wildlife managers. While our study demonstrates the suitability of CKMR for small caribou populations using samples collected from a single, non‐invasive sampling event, whether these cost savings are realized largely will depend upon the sampling effort required to obtain a sufficient number of kin pairs to achieve the desired levels of accuracy and precision. Therefore, we caution managers to carefully consider their goals and objectives, as well as their available resources (personnel, time, money, etc.) and the particularities of their system before deciding whether a CKMR‐based method (whether from a single sampling event or multiple sampling events) is the appropriate choice for their management needs.

## AUTHOR CONTRIBUTIONS

**Brandon D. Merriell:** Conceptualization (equal); formal analysis (lead); writing – original draft (lead); writing – review and editing (equal). **Micheline Manseau:** Conceptualization (equal); supervision (equal); writing – review and editing (equal). **Paul J. Wilson:** Conceptualization (equal); supervision (equal); writing – review and editing (equal).

## CONFLICT OF INTEREST STATEMENT

The authors declare no competing interests.

## Data Availability

The data and code that support the findings of this study are publicly available in Dryad at https://doi.org/10.5061/dryad.jh9w0vtk2 but will be made publicly available upon acceptance for publication.

## Appendix

**TABLE A1:** Input settings used for COLONY parentage assignment.

Mating system I	Female polygamy
	Male polygamy
Mating system II	With inbreeding
	Without clone
Species	Dioecious
	Diploid
Length of run	Long
Analysis method	Full‐likelihood (FL)
Likelihood precision	Very high
Run specifications	Update allele frequency: No
	Sibship scaling: No
	Number of runs: 5
	Random number seed: 1234
Sibship prior	No prior
